# Tailoring the gelatin/chitosan electrospun scaffold for application in skin tissue engineering: an in vitro study

**DOI:** 10.1007/s40204-018-0094-1

**Published:** 2018-08-23

**Authors:** Mohamad Pezeshki-Modaress, Mojgan Zandi, Sarah Rajabi

**Affiliations:** 1 Burn Research Center, Iran University of Medical Sciences, Tehran, Iran; 2 Department of Biomaterials, Iran Polymer and Petrochemical Institute, P.O. Box: 14965/159, Tehran, Iran; 3 Department of Stem Cells and Developmental Biology, Cell Science Research Center, Royan Institute for Stem Cell Biology and Technology, ACECR, P.O. Box 19395/4644, Tehran, Iran

**Keywords:** Gelatin/chitosan, Blend ratio, Nanofibers, Skin, HDF cells, In vitro

## Abstract

**Abstract:**

The nanofibrous structure containing protein and polysaccharide has good potential in tissue engineering. The present work aims to study the role of chitosan in gelatin/chitosan nanofibrous scaffolds fabricated through electrospinning process under optimized condition. The performance of chitosan in gelatin/chitosan nanofibrous scaffolds was evaluated by mechanical tests, scanning electron microscopy (SEM), Fourier transform infrared (FTIR) and in vitro cell culture on scaffolds with different gelatin/chitosan blend ratios. To assay the influence of chitosan ratio on biocompatibility of the electrospun gelatin/chitosan scaffolds for skin tissue engineering, the culturing of the human dermal fibroblast cells (HDF) on nanofibers in terms of attachment, morphology and proliferation was evaluated. Morphological observation showed that HDF cells were attached and spread well on highly porous gelatin/chitosan nanofibrous scaffolds displaying spindle-like shapes and stretching. The fibrous morphologies of electrospun gelatin/chitosan scaffolds in culture medium were maintained during 7 days. Cell proliferation on electrospun gelatin/chitosan scaffolds was quantified by MTS assay, which revealed the positive effect of chitosan content (around 30%) as well as the nanofibrous structure on the biocompatibility (cell proliferation and attachment) of substrates.

**Graphical abstract:**

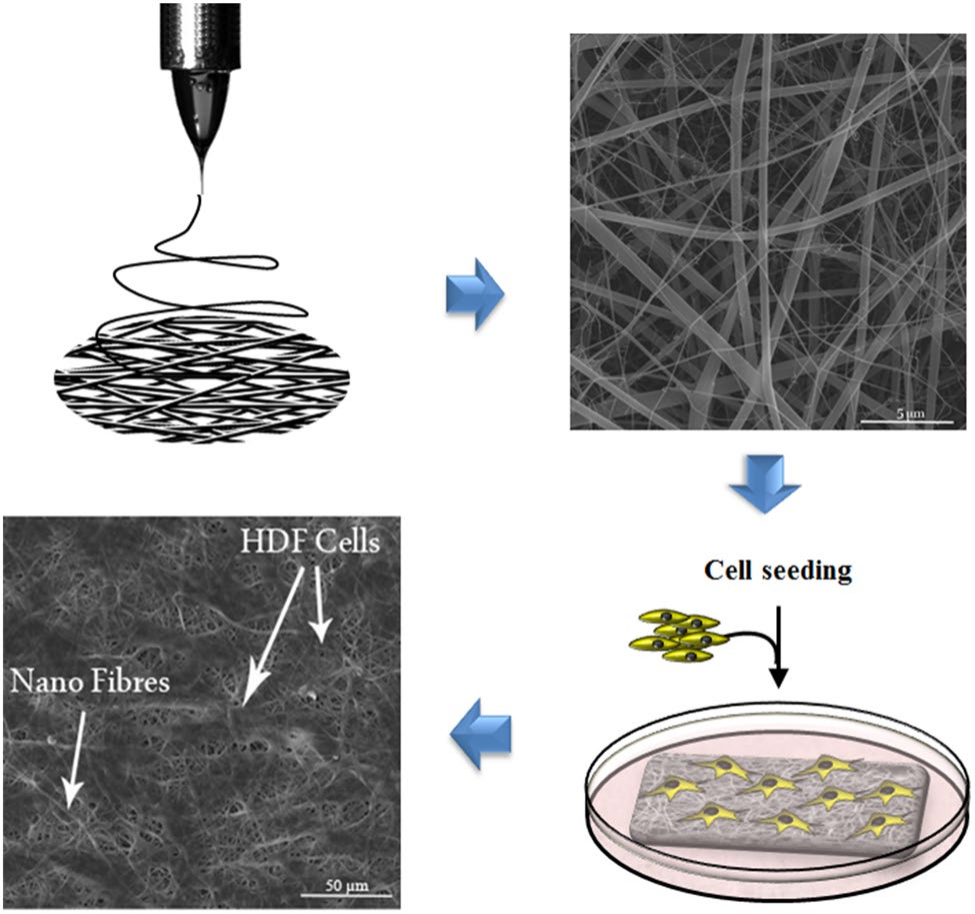

## Introduction

In recent years, electrospinning as a reliable technique for production of biomimetic scaffolds containing large network of interconnected pores has gained great attention in the literature (Bhardwaj and Kundu 2010; Dabouian et al. 2018; Pezeshki-Modaress et al. 2014; Saeed et al. 2017).

The human body tissue is composed of cells and extracellular matrix (ECM) which provide proper structural components as well as controlling the body processes, performances and wound healings (Sell et al. 2010). The ECM contains highly hydrated macromolecular networks such as collagen and glycosaminoglycans (Wang et al. 2007). Tissue engineering provides constructs appropriate for tissue substitution. A crucial factor in tissue engineering is to design and fabricate a biocompatible and biodegradable scaffold for culturing or hosting cells and transplanting into the body to regenerate the neo-organs (Pietrucha and Marzec 2005).

The cells have to interact with the scaffolds’ structure in three dimensions. In natural ECM structure protein fibers’ diameters are smaller than the cells and could provide a direct contact with the cells in three-dimensional orientations. In summary, the tissue-engineered scaffold should provide the opportunity for to exchange the signals between cells and the microenvironment and also between the cells in regeneration process (Barnes et al. 2007). Therefore, electrospunnanofibrous substrates are good candidates for using as tissue-engineered scaffolds with nano-scale structure (Heydarkhan-Hagvall et al. 2008). Many research works have focused on proteins as biopolymers for fabrication of nanofibrous scaffolds. The components of natural tissues, collagen and GAGs are widely used for scaffold fabrication which serves as efficient substitutes for native ECM (Mottaghitalab et al. 2015; Zhong et al. 2005). Gelatin is a natural biopolymer which is notably similar to collagen and still less susceptible to degradation during electrospinning process and enjoy a great potential to conduct the migration, adhesion, growth and organization of cells during regeneration process (Heydarkhan-Hagvall et al. 2008; Mahboudi et al. 2015; Pant and Kim 2013; Pezeshki-Modaress et al. 2013; Sadeghi et al. 2018; Zandi et al. 2007). Chitosan including glucosamine and *N*-acetylglucosamine is a biocompatible and biodegradable polymer and in vivo assays have proven that chitosan-based biomaterials show non-inflammatory reaction after injection, implantation and ingestion in the human body (Barikani et al. 2014; Baxter et al. 2013; Jayakumar et al. 2011; Mao et al. 2003a). Scaffolds containing chitosan also benefit other useful properties such as wound healing property (because of structural similarity to glycosaminoglycans), reducing scars, hemostasis, antifungal and bacteriostasis character, which make it applicable as a dermal scaffold. Therefore, using the blend based on gelatin and chitosan to improve their individual properties could be applicable as scaffolding materials in tissue regeneration (Esfandiarpour-Boroujeni et al. 2016; Martínez-Camacho et al. 2011; Modaress et al. 2012; Pezeshki-Modaress et al. 2013; Rahman et al. 2013). It has been reported that a higher ratio of gelatin (> 50% w/w) in the gelatin/chitosan blended scaffolds resulted in better cell attachment and proliferation by considering the literature (Jafari et al. 2011; Modaress et al. 2012; Pezeshki-Modaress et al. 2013), but to the best of our knowledge there is no study on the influence of chitosan ratio on the nanofibrous scaffold properties in the literature. TFA/DCM (70/30) solvent system has been introduced as applicable solvent for electrospinning of gelatin/chitosan blends (Dhandayuthapani et al. 2010). Jafari et al. have fabricated gelatin/chitosan electrospun nanofibers using low molecular weight chitosan (*M*_w_ 1000 g mol^−1^) and exhibited the potential of produced nanofibers for skin tissue engineering (Jafari et al. 2011). Pezeshki et al. introduced the optimized conditions for electrospinning process of gelatin/chitosan at TFA/DCM (70/30) solvent system using response surface methodology (Pezeshki-Modaress et al. 2014). In this work, nanofibrous structures of gelatin/chitosan blends were fabricated and the effects of chitosan ratio (30, 40, 50 w/w) on the chemical, physical and biological property of the obtained nanofibers were studied.

## Experimental

### Materials

Gelatin type B and chitosan of medium weight were purchased from Sigma-Aldrich (USA). *N*-(3-dimethylaminopropyl)-*N’*-ethylcarbodiimide hydrochloride (EDC), trifluoroacetic acid (TFA), dichloromethane (DCM) and ethanol were purchased from Merck (Germany). DMEM/F12 medium, FBS, trypsin/EDTA, l-glutamine, and penicillin/streptomycin were purchased from Gibco, Canada.

### Preparation of polymer solution

Chitosan solution of 5% (w/v) and gelatin solution of 15% (w/v) were prepared by dissolving them in a co-solvent system of TFA and DCM (70:30) as previously reported (Pezeshki-Modaress et al. 2014). The two solutions were agitated overnight at room temperature to achieve homogeneous solutions with different ratios of 50/50, 60/40 and 70/30 (gelatin/chitosan). The solution was poured into a 5 mL syringe and was subjected to the electrospinning process using a horizontal system with a cylindrical collector covered by aluminum foil (Co881007 NYI, ANSTCO, Iran) at 30 °C. Electrospinning was performed at 27 kV applied voltage and 0.5 mL/h flow rate. The distance between the needle tip and collector was 100 mm. The electrospun nanofibers were kept at 4 °C and dry condition until further characterization.

### Morphologies of the fibers

The morphology and diameters of electrospun nanofibers of gelatin/chitosan were investigated using scanning electron microscopy (SEM, VEGA, TESCAN, Czech) after gold sputter coating. The diameter of electrospun nanofibers was measured using image analysis software (Image J 1.42q, National Institute of Health, USA). At least 200 different fibers were used to determine the MFD and SDF.

### Measurement of porosity

The average porosity of the electrospun gelatin/chitosan samples with different compositions was measured by liquid displacement technique (Jiankang et al. 2007; Modaress et al. 2012; Pezeshki-Modaress et al. 2017). Ethanol was selected as the displacement liquid since it permeates through the scaffolds without swelling or shrinking the matrix. The scaffolds (dry weight, *w*_d_) were immersed in ethanol for 30 min and the weights of the scaffolds in ethanol were recorded as *w*_l_. The liquid on the surface of the scaffolds was removed by filter paper after taking out ethanol. The weight of the wet scaffolds was recorded as *w*_w_. The porosity of the gelatin/chitosan scaffolds was obtained by

$${\text{Porosity }}\left( \% \right) = \left( {W_{\text{w}} - W_{\text{d}} } \right)/\left( {W_{\text{w}} - W_{ 1} } \right) \, \times { 1}00$$

The values are expressed as the means ± standard error (*n* = 3).

### Mechanical properties

The tensile mechanical properties were tested with a mechanical tester (SANTAM, STM-20, Iran). The samples were rectangular disks (30 × 10 mm^2^) with a thickness of around 40 µm tested at a constant tensile deformation rate of 5 mm/min in the dry state at room temperature. The stress and elongation-at-break were determined. The values are expressed as the means ± standard error (*n* = 3) (Mao et al. 2003b; Modaress et al. 2012).

### Fourier transform infrared (FTIR) analysis

The chemical structure of the gelatin/chitosan nanofibers was analyzed by Fourier transform infrared spectroscopy (FTIR) using a BRUKER FTIR spectrophotometer (EQUINOX 55, Germany). The infrared spectra of the samples were measured over a wavelength range of 4000–400 cm^−1^.

### Crosslinking and sterilization

The nanofibrous scaffolds were chemically crosslinked using 0.02 g *N*-(3-dimethylaminopropyl)-*N’*-ethylcarbodiimide hydrochloride (EDC) (Merck, Germany) in 10 mL pure ethanol for 24 h, and then sterilized with 70% ethanol for 4 h. They were rinsed several times in phosphate buffer solution (PBS) to remove traces of ethanol. The cross-linked electrospun scaffolds were kept at 4 °C under dry condition for further assessment.

### In vitro human dermal fibroblast cell culture

HDF cells were cultured in DMEM/F12 medium containing 10% fetal bovine serum (FBS), 1% penicillin/streptomycin and 1% l-glutamine in T-75 flask tissue culture. Four and six passage cells were used in all the experiments. The cells were cultured at 37 °C and 5% CO_2_. The culture medium was refreshed every 72 h. At confluence, fibroblast cells were harvested and subcultivated in the same medium. The cells were separated with 0.05% trypsin/EDTA, centrifuged, and re-suspended in medium. The sterile nanofibrous scaffolds were incubated in culture medium overnight in order to check contamination, increase protein adsorption and cell attachment onto the nanofibers. The density of 1 × 10^4^ cells/cm^2^ in culture medium (DMEM/F12, Gibco, Canada), containing 10% fetal bovine serum, was seeded on the scaffold in a 24-well culture plates. The medium was replaced regularly every 48 h; the culture process was carried out in an incubator at 37 °C with 5% CO_2_. All experiments were run in triplicate. Cell proliferation on film served as reference and control substrates. The samples were analyzed by DAPI staining, MTS and scanning electron microscopy (SEM).

### MTS Assay

To evaluate the cell proliferation and metabolic activity on scaffolds, the MTS (Promega, G5421) assay was performed according to the manufacturer’s instructions. Briefly, HDF cells were seeded at a density of 1 × 10 ^4^ cells/cm^2^ on scaffolds. The medium was changed every 2 days. At days 1, 3, 7 and 14, the scaffolds were transferred into new wells and the MTS solution was added into each well, after which the plates were incubated in the dark at 37 °C for 3 h. The absorbance of the solution was measured at 490 nm. The experiments were run in triplicate.

### Scanning electron microscopy and DAPI staining

As far as the study of the morphological characteristics of cells cultured onto the nanofibrous matrices and also maintaining the fibrous structure of gelatin/chitosan substrate at cell culture medium were concerned, SEM observations were carried out. The morphological characteristics of the cells cultured onto the nanofibrous matrices were studied through scanning electron microscopy. After growing for 1 and 7 days, the cellular constructs of the HDF cells were harvested, washed with PBS to remove non-adherent cells and then fixed with 2.5% glutaraldehyde overnight at 4 °C, dehydrated through a series of graded alcohol solutions (50, 70, 80, 90, 95 and 100%) and then vacuum-dried overnight. Dry cellular constructs were sputter coated with gold and observed by SEM at an accelerating voltage of 15 kV. For DAPI staining process, samples were fixed for 2 h in a 10% PBS/neutral-buffered formalin solution (pH 7.4) at 25 °C. Subsequently, samples were washed in d.d.H_2_O and dehydrated in a graded alcohol series. Then, the samples were stained with 4,6-diamidino-2-phenylindole (DAPI, Sigma-Aldrich, D8417), after which they were visualized utilizing an Olympus fluorescent microscope (BX51 with Olympus DP72 digital camera).

## Results and discussion

### Morphology of the scaffolds

**Fig. 1:**
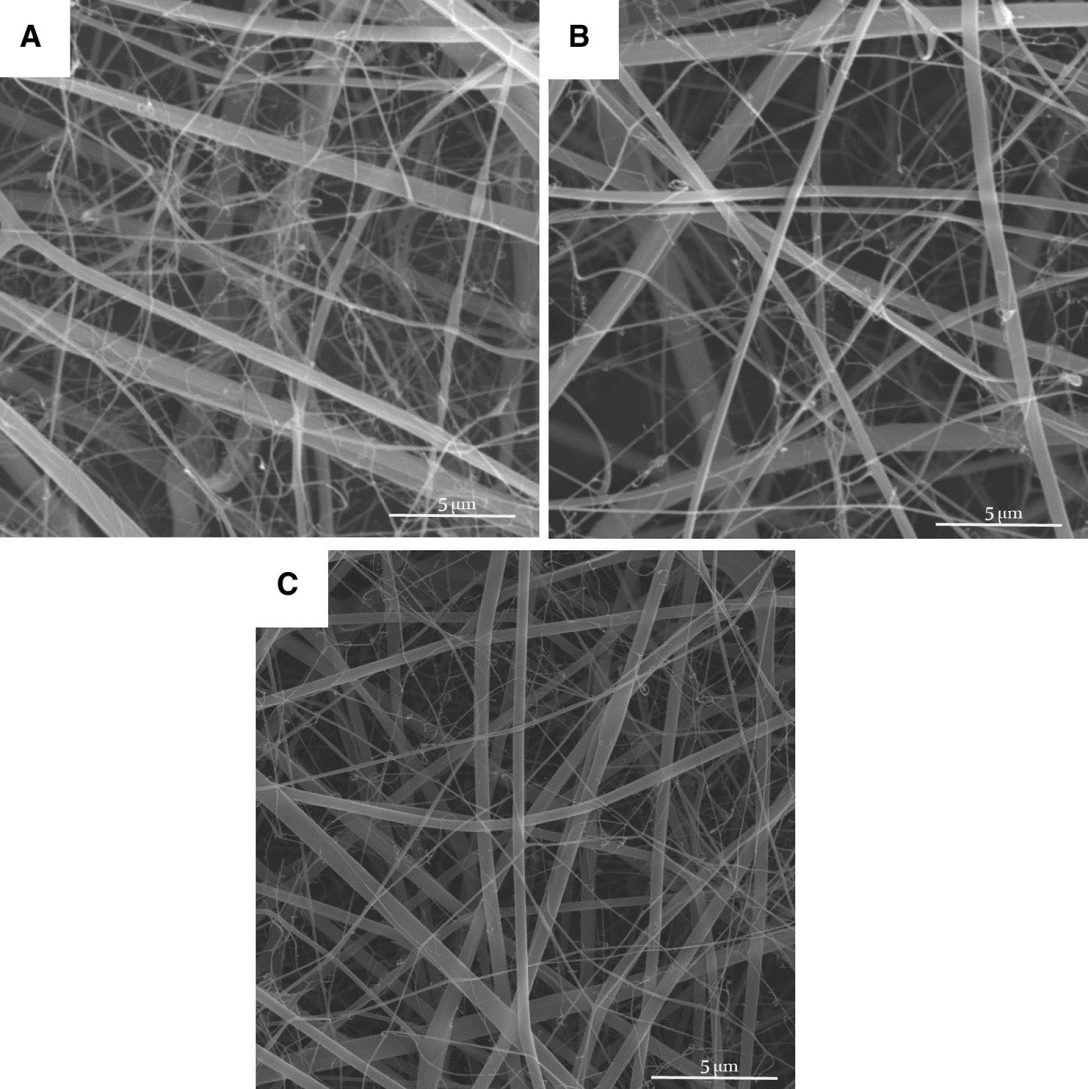
SEM micrographs of nanofibrous scaffolds fabricated using 0.5 mL/hr flow rate and 27 kV applied voltage. **a** Chi 50, **b** Chi 40, **c** Chi 30

The nanofibrous structure of gelatin/chitosan blends scaffolds with suitable properties was prepared using electrospinning technique. Scaffolds with four different gelatin/chitosan blend ratios of 100/0, 70/30, 60/40 and 50/50 were fabricated and the influence of chitosan ratio on chemical, physical, mechanical and biological properties of the scaffolds was investigated. Figure 1 shows SEM micrographs of the electrospun gelatin/chitosan scaffolds containing different ratio of chitosan. All scaffolds have nanofibrous structure and no beads were observed. The mean fiber diameter and their standard deviation of fabricated scaffolds are listed in Table 1.

**Table 1:** The MFD and SDF of electrospun gelatin/chitosan scaffolds

Sample	MFD (nm)	SDF (nm)
Chi 50	180	190
Chi 40	196	185
Chi 30	185	180

### Physical and mechanical properties of gelatin/chitosan scaffolds

**Fig. 2:**
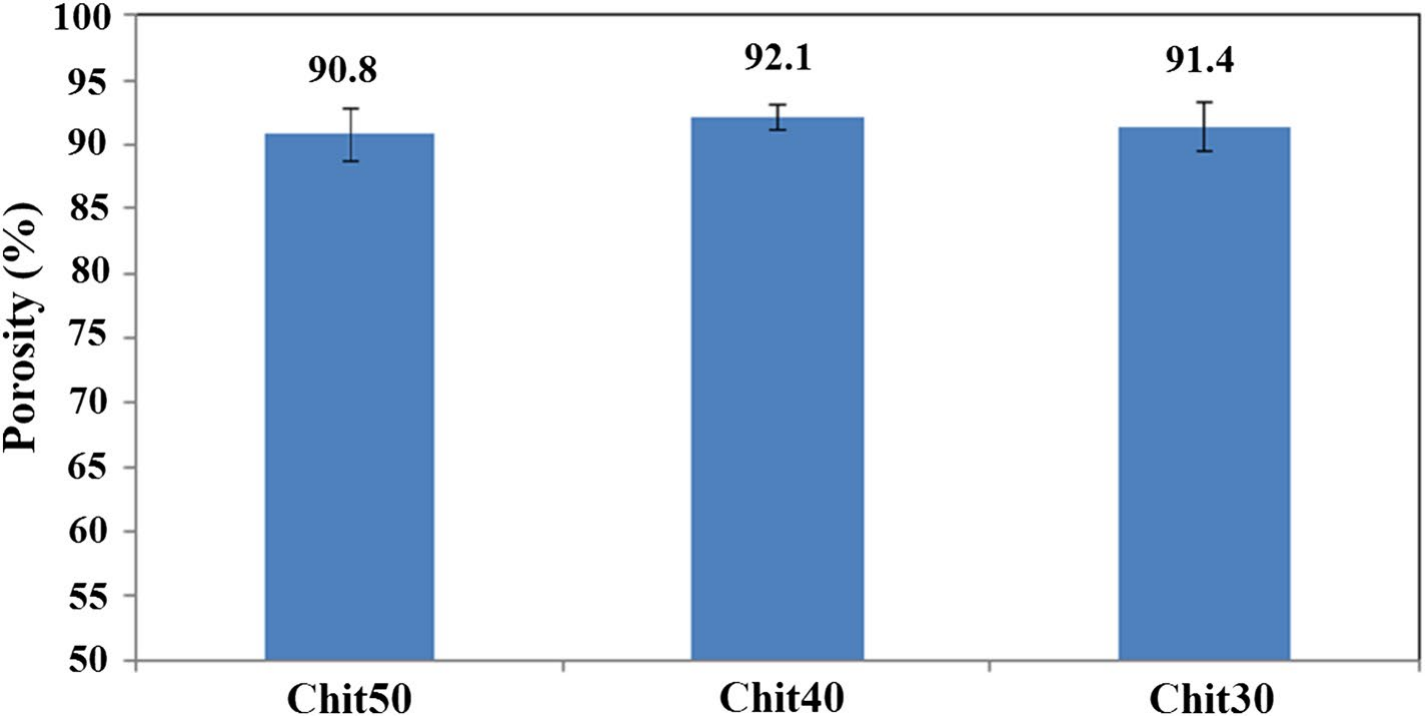
The porosity of electrospun scaffolds containing different ratio of chitosan

The dermis regeneration process would be achieved when nutrients penetrate into the scaffold through interconnected pores and the exudation of the wound can be steadily absorbed by the scaffold. The structures with high porosity and pore interconnectivity not only improve mass transfer of oxygen and nutrients into the inner pores, but also efficiently remove metabolic products (Jiankang et al. 2007; Pezeshki-Modaress et al. 2013). The porosity of the electrospun gelatin/chitosan scaffolds containing different ratios of chitosan is illustrated in Fig. 2. The preferred porosity of scaffolds used for tissue engineering should generally be within the range of 60–90% (Chandrasekaran et al. 2011; Chong et al. 2007). The porosity of the electrospun gelatin/chitosan scaffolds achieved in this study was around 90%, comparable to the scaffold fabricated from conventional scaffolding techniques such as phase separation, salt leaching and fiber bonding and also at preferred porosity range of scaffolds used for tissue engineering (Chandrasekaran et al. 2011; Chong et al. 2007). One of the main advantages of the electrospun nanofibrous scaffolds is their highly interconnected pores which are created by nanofibers lying loosely upon each other. The nanofibrous network structure fabricated using electrospinning technique possess interconnected pores, which are created by nanofibers lying loosely upon each other, best mimics the natural ECM and improves its application as tissue engineering scaffolds. The obtained results reveal that all the prepared electrospun samples are highly porous and there is no significant difference between the electrospun samples.

**Fig. 3:**
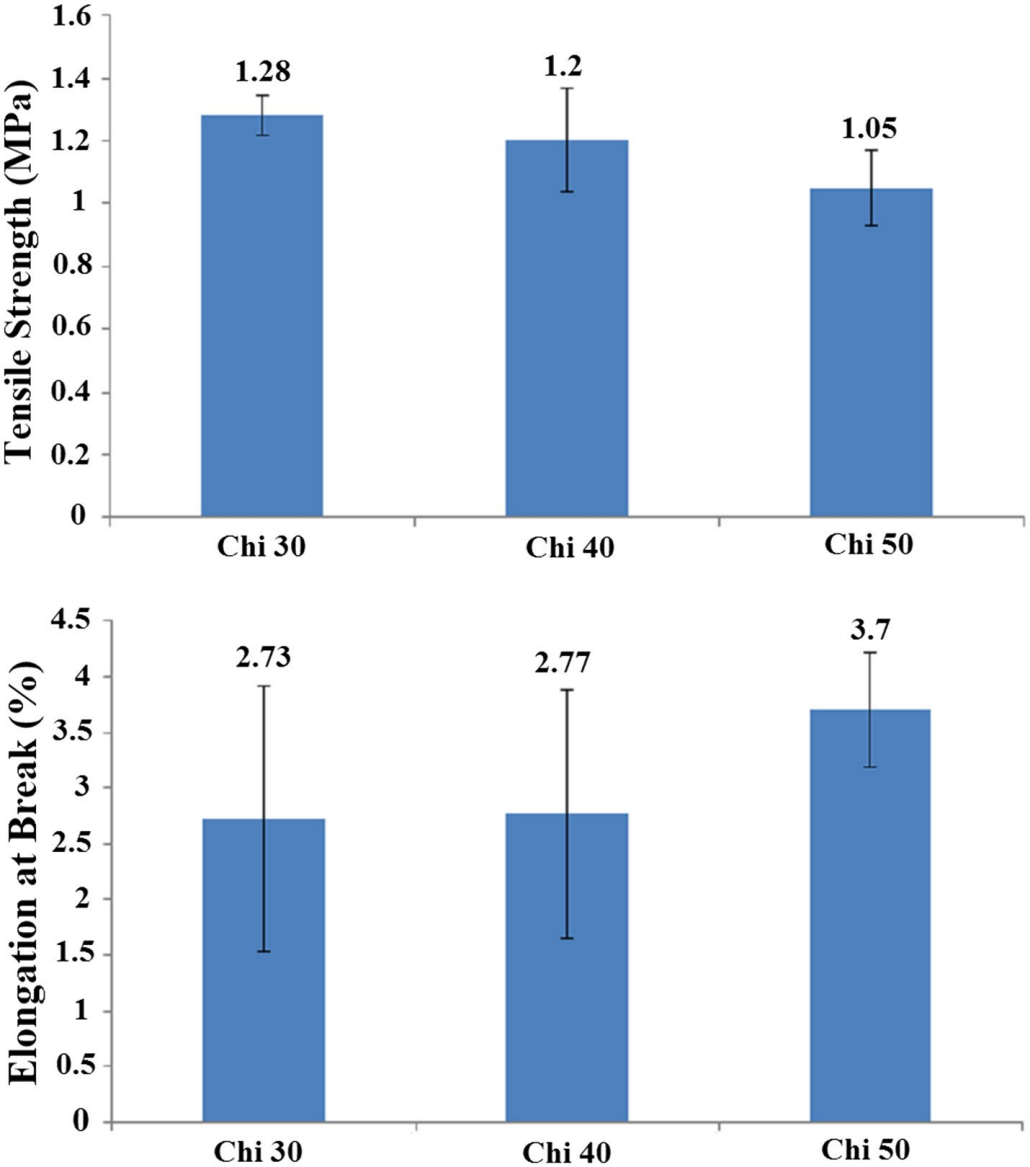
The mechanical property of gelatin/chitosan electrospun scaffolds

The high degree of porosity and interconnected pore morphology (as illustrated in Figs. 1, 2) of electrospun scaffolds fabricated in this study could provide good nutrient and oxygen transfer throughout the porous scaffolds, which results in better cell migration and regulating ECM formation. The mechanical performance of scaffolds in skin tissue engineering, subjected to tensile stresses and has high area/thickness ratio, is an important factor that can influence the clinical operation and wound healing. The mechanical property of nanofibrous scaffolds with different chitosan ratios is illustrated in Fig. 3. The results reveal that the tensile strength and elongation-at-break of all three nanofibrous scaffolds were more than 1 MPa and 2.7%, respectively. The same as porosity, the mechanical property of three samples was not statistically different.

### Chemical analysis

**Fig. 4:**
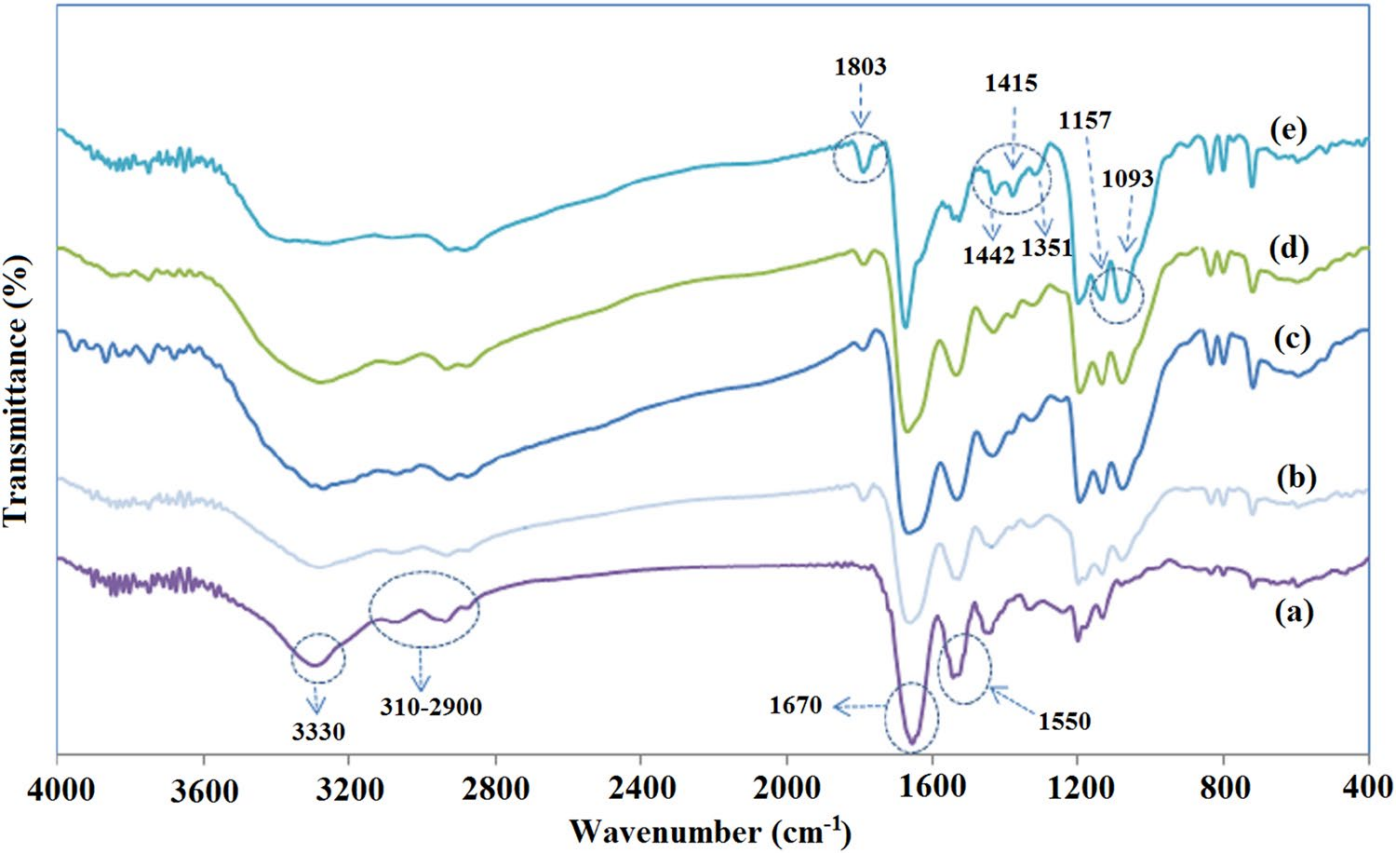
The FTIR analysis of gelatin/chitosan electrospun scaffolds containing different ratio of chitosan: a) Chi 0 b) Chi 30 c) Chi 40 d) Chi 50 e) Chi 100

The chemical structure of fabricated gelatin/chitosan nanofibers was investigated using FTIR spectroscopy analysis. Representative spectra for blended samples, as well as gelatin (Chi 0) and chitosan (Chi 100) electrospun nanofibers in the wave number range of 400–4000 cm^−1^, are shown in Fig. 4. All nanofibers’ spectra exhibited peaks at 3330 cm^−1^ for –NH_2_ and –OH stretching vibration and 3100–2900 cm^−1^ for C–H aliphatic group stretching vibration (3100 cm^−1^ for alkenyl C–H stretch and 2970 cm^−1^ for CH_2_ asymmetrical stretching).

The electrospun gelatin (Chi 0) spectrum illustrated several characteristic absorption bands at 1670 cm^−1^ for amid 1 (C=O) stretching vibration, 1550 cm^−1^ for amid 2 (N–H) bending vibration, 1465 cm^−1^ for CH_2_ bending, 1262 cm^−1^ for amid 3 (C–N) stretching vibration and 1160 cm^−1^ for –C–O stretching (Al-Saidi et al. 2012; Bin Ahmad et al. 2011; Lai et al. 2012; Nguyen and Lee 2010). The electrospun chitosan (Chi 100) spectrum exhibited strong peak at 1680 cm^−1^ with a shoulder around 1645 cm^−1^ and a peak at 1566 cm^−1^ for C=O stretching vibration, vibration of amine group and ammonium ions, respectively (Haider et al. 2010; Qian et al. 2011). The peak at 1803 represents CO–F group. The Chi 100 spectrum displayed also some peaks at 1442, 1415 and 1351 cm^−1^ for C–H bending vibration, 1093 and 1157 cm^−1^ for C–O stretching vibration (Bin Ahmad et al. 2011; Dhandayuthapani et al. 2010; Yin et al. 2003).

The FTIR spectra of the nanofibrous gelatin/chitosan blends exhibited characteristic absorption bands at 1680 and 1554 cm^−1^ representing the carboxyl and amine groups.

The shifting and broadening of both 1680 and 1554 cm^−1^ peaks for gelatin/chitosan blend spectrum in comparison of pure gelatin and chitosan spectrum revealed the formation of hydrogen bonding between chitosan and gelatin. The hydroxyl, carboxyl and amine groups of gelatin could form hydrogen bond with hydroxyl and amine groups of chitosan. These interactions could lead to polyanionic–polycationic complex (Bin Ahmad et al. 2011; Dhandayuthapani et al. 2010; Qian et al. 2011). The gelatin/chitosan blends spectrum show absorption bands at 1093 and 1415 cm^−1^ representing the C–O stretching (represent the saccharide structure of chitosan) and C–H bending vibration, respectively, which is absent in pure gelatin spectrum. There is another important peak at 1803 cm^−1^ in Chi 100 and gelatin/chitosan blends spectrum for CO–F groups which is absent in pure gelatin spectrum.

### In vitro cell adhesion and proliferation

**Fig. 5:**
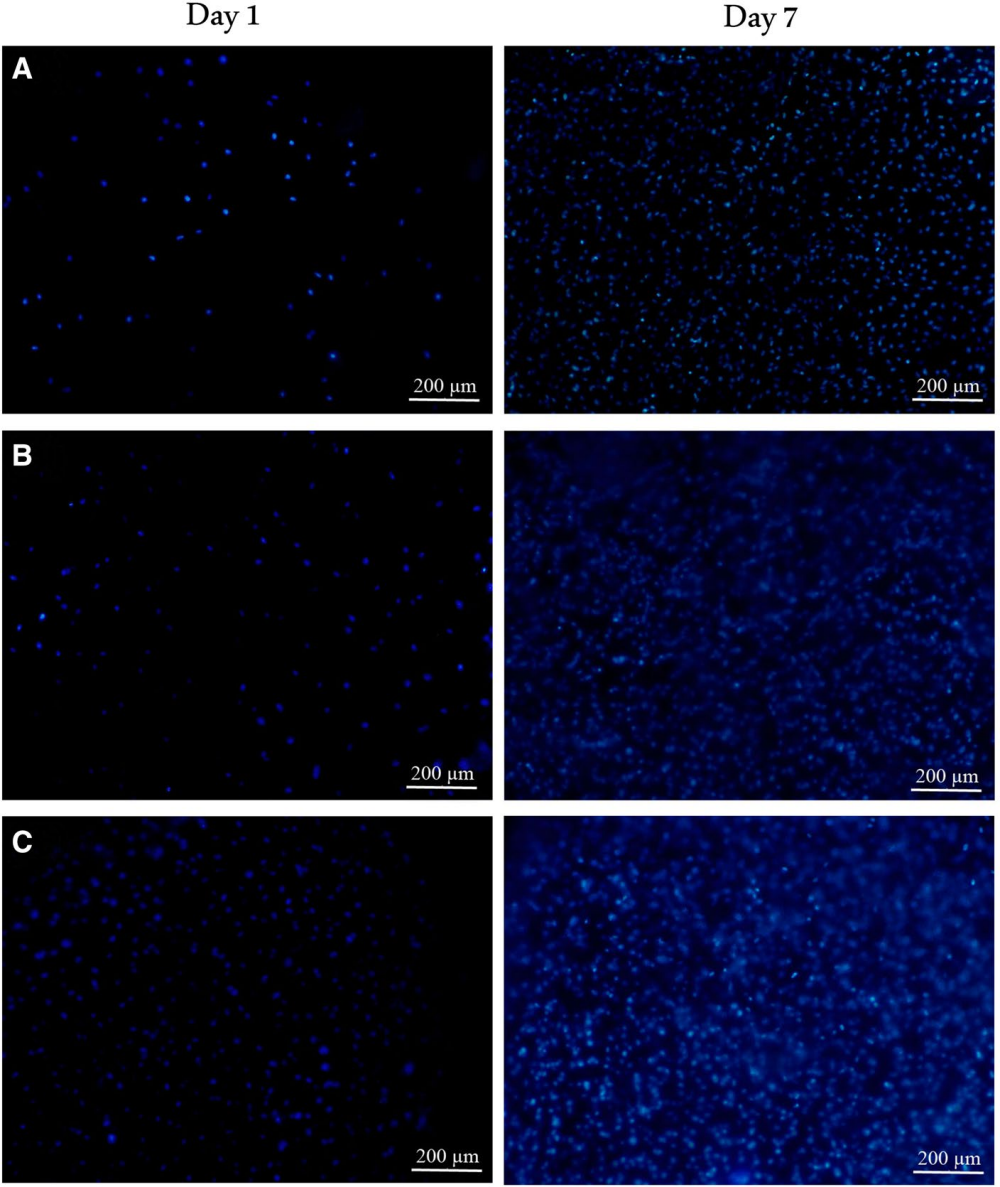
DAPI staining of HDF cells on electrospun gelatin/chitosan scaffold after 1 and 7 days of culturing. **a** Chi 50 **b** Chi 40 **c** Chi 30

**Fig. 6:**
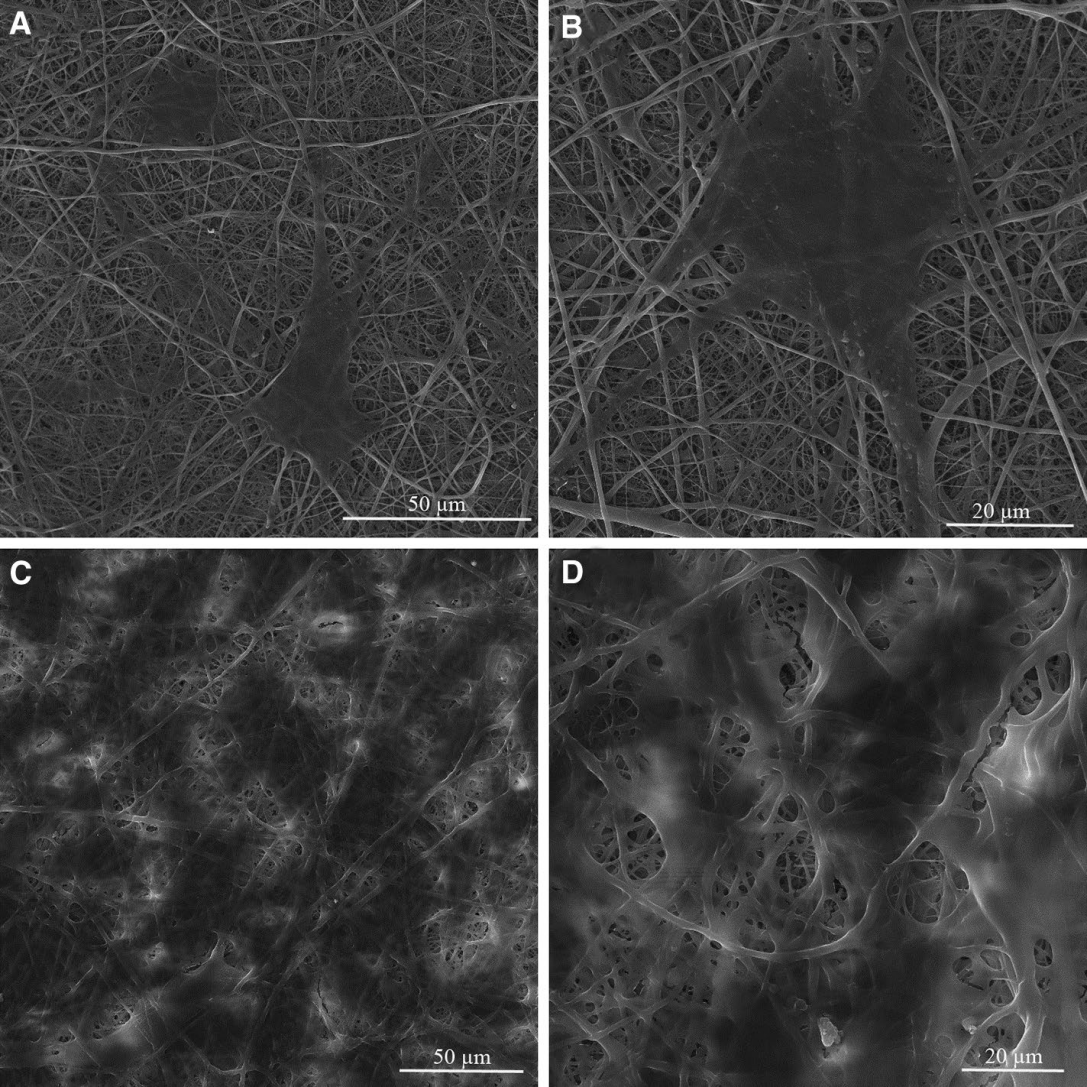
Morphology of HDF cells on electrospun Chi 50 scaffold after 1 (**a**, **b**) and 7 (**c**, **d**) days of culturing with different magnification

**Fig. 7:**
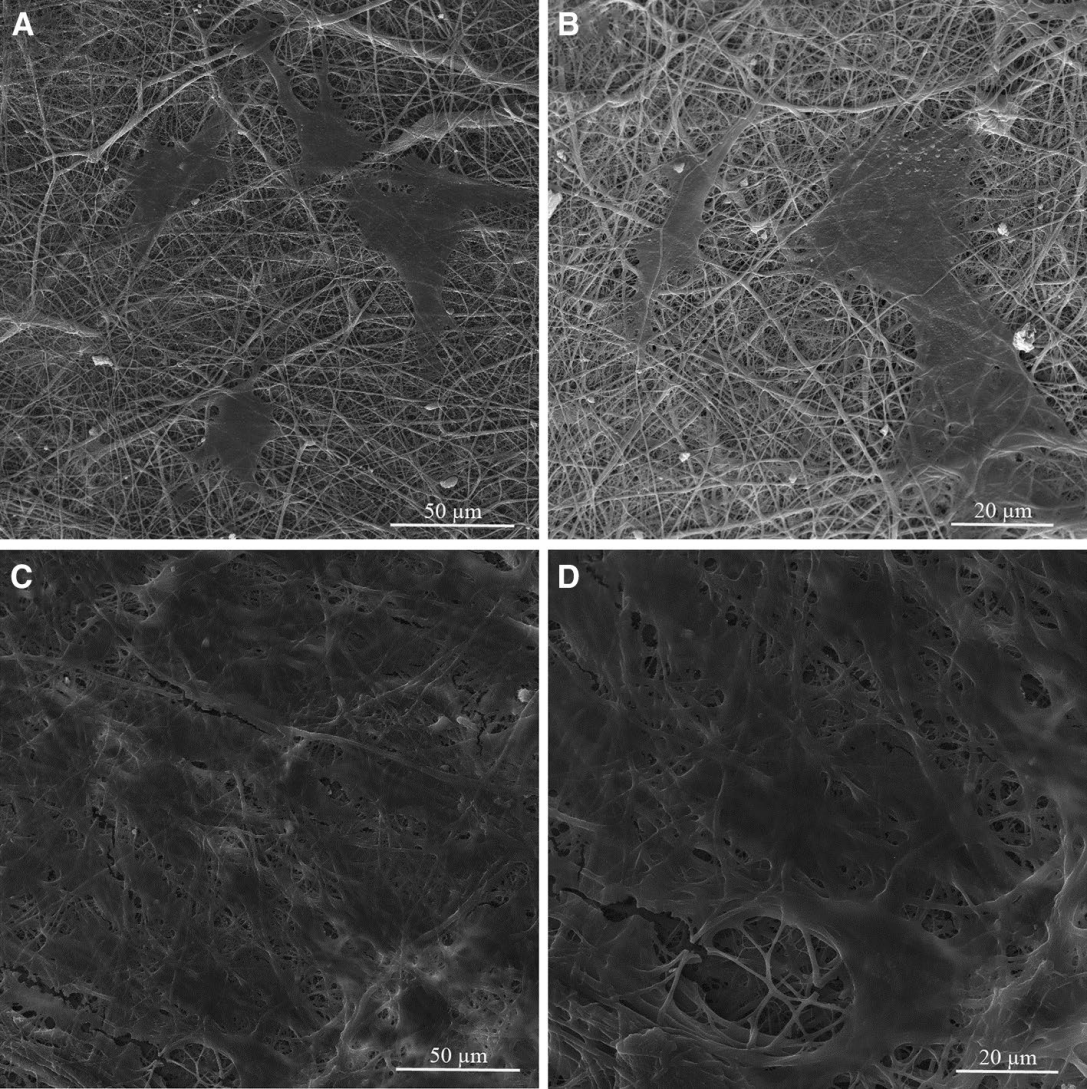
Morphology of HDF cells on electrospun Chi 40 scaffold after 1 (**a**, **b**) and 7 (**c**, **d**) days of culturing with different magnification

**Fig. 8:**
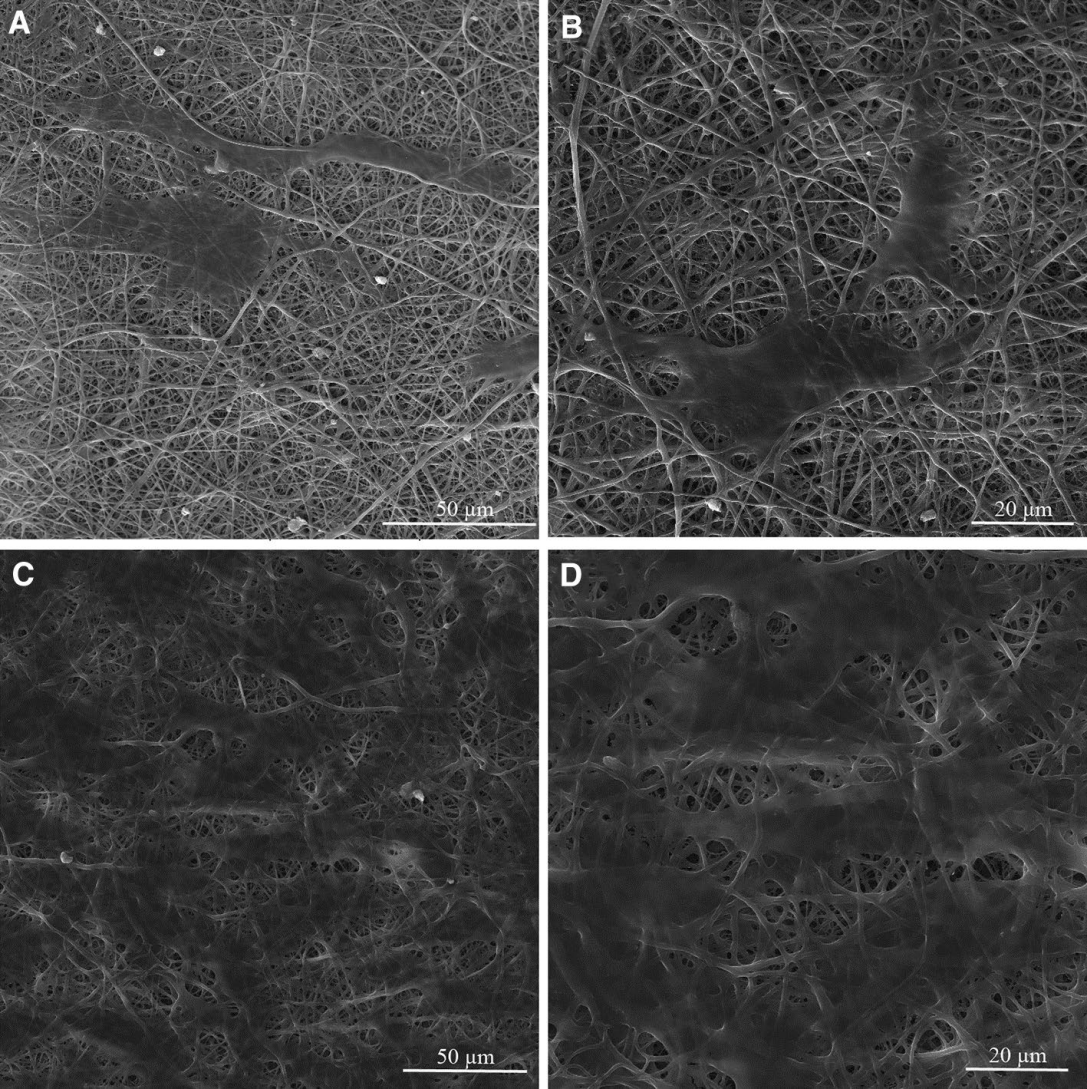
Morphology of HDF cells on electrospun Chi 30 scaffold after 1 (**a**, **b**) and 7 (**c**, **d**) days of culturing with different magnification

**Fig. 9:**
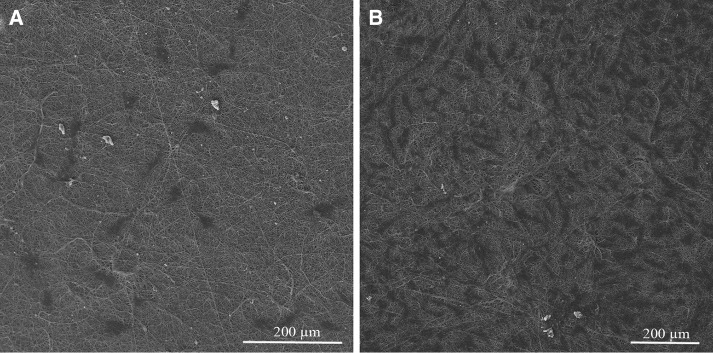
Micrograph of HDF cells on electrospun Chi 30 scaffold after 1 (**a**) and 7 (**b**) days of culturing with different magnification

The cell biocompatibility of the gelatin/chitosan nanofibrous scaffolds was investigated in vitro by inspecting the adhesion, spreading and proliferation of HDF cells onto electrospun scaffolds. HDF Cells bioactivity on nanofibers in terms of attachment and proliferation are fundamental to evaluate  nanofibers capability as promising scaffolds which is achieved using DAPI staining, SEM observation and MTS assay. The DAPI staining results are shown in Fig. 5 at days 1 and 7, which reveal the good attachment and proliferative behavior of cells on all three nanofibrous scaffolds; however, the Chi 50 shows lower cell attachment in comparison with other samples. The SEM observation was used to investigate the cell interaction accurately with the nanofibrous substrates at day 1 and 7 to fulfill the DAPI staining results. Figures 6, 7 and 8 represent the micrographs of HDF cells cultured on electrospun scaffolds containing different ratios of chitosan which display the high HDF cells attachment and well spreading on all three nanofibrous scaffolds with spindle-like shape and complete stretching morphology. Comparison of micrographs at days 1 and 7 reveals the population of cells and shows consistency with DAPI staining results. The results of SEM observation at day 1 reveal that the HDF cells interacted well with the surrounding fibers and attached to the surfaces byfilopodia. The HDF cells adhered on gelatin/chitosan nanofibers and demonstrated the characteristic spindle-like shape of HDF cells, signifying that the gelatin/chitosan nanofibers maintained the phonotype of fibroblast cells. The neighbor cells were interconnected by excreted filopodia which could be observed in SEM micrographs. The observation at day 7 reveals the cell migration, proliferation and also the formation of a continuous monolayer covering the surface of nanofibers by interconnection of adjacent cells. Comparison of Fig. 9a, b clearly displays the population of cells during 7 days on Chi 30. These results also demonstrated that electrospun gelatin/chitosan scaffolds have maintained its nanofibrous morphology for 7 days at cell culture media employing suitable crosslinking conditions. Because of discrepancy in the hydrophilicity, chemical composition and charge of proteins such as gelatin and polysaccharides like chitosan (Chen et al. 2010; Pezeshki-Modaress et al. 2013; Zhong et al. 2005), electrospun gelatin/chitosan scaffolds containing different amount of chitosan (as a positively charged polysaccharide) were expected to reveal different cellular behavior. The HDF cell proliferation on electrospun gelatin/chitosan nanofibrous scaffolds was quantified by MTS assay for 14 days (1, 3, 7 and 14 days). The electrospun gelatin (Chi 0) was used as control for evaluating the role of chitosan in the HDF cellular activity. To investigate the influence of substrate nanotopography on cells population, we have used a gelatin/chitosan cast film as second control which was fabricated from the same solution that was used for the preparation of Chi 40 sample.

**Fig. 10:**
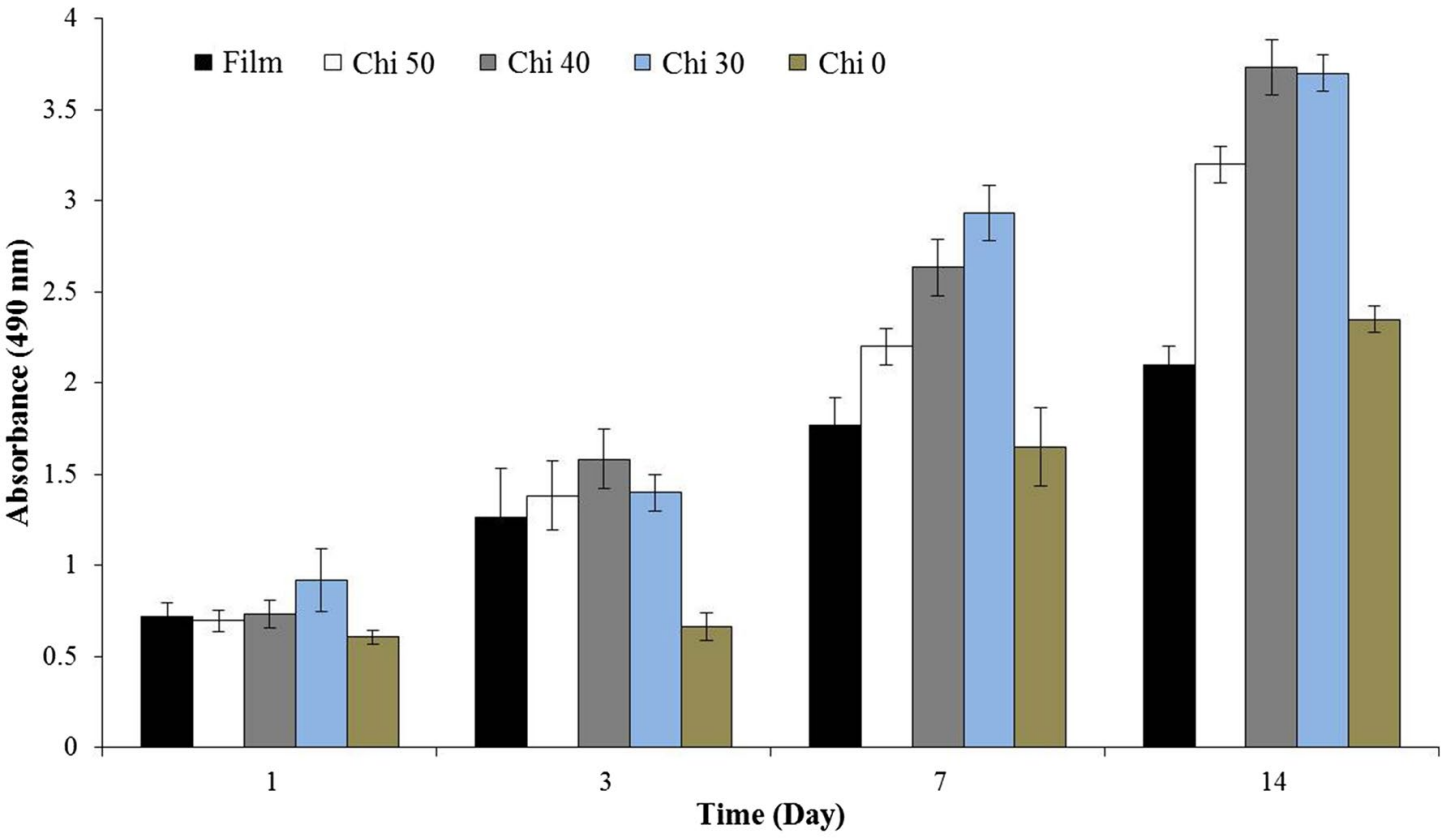
MTS assay after HDF cells were cultured on nanofibrous scaffolds. Formazan absorbance expressed as a function of culture time

As shown in Fig. 10, in 14 days of HDF cells culturing, the cell number increased with culture time on all substrates groups. On days 1 and 3, no statistically difference in proliferation of cells on the substrates could be observed except the Chi 0 which exhibited low cell proliferation. At 7 and 14 days, the substrates performance for proliferation of HDF cells could be observed more clearly. The nanofibrous scaffolds Chi 40 and Chi 30 show the best performance for HDF cell proliferation. When the cell proliferation between the Chi 40 electrospun scaffold and gelatin/chitosan (60/40)cast film was compared, the HDF cells response much better on the nanofibrous scaffold which reveal the potential of electrospinning method. When the cell proliferation of the Chi 0 nanofibrous scaffold is compared with Chi 30, 40 and 50 nanofibrous scaffolds, it is confirmed that the chitosan presence in the gelatin structure has improved the proliferation rate of HDF cells. Our results demonstrated that using nanofibrous structure and the presence of polysaccharide molecules (chitosan) in blend with gelatin would be useful for cellular behavior of substrates. Moreover, the presence of 40% polysaccharide in composition of blends shows higher influence than substrate morphology on the cells proliferation considering the lowest cells proliferation for Chi 0. These results signify that there is a delicate relationship between chitosan and gelatin ratio for responding to the HDF cells. It seems that the low chitosan containing scaffolds (Chi 30) might have accelerated adhesion and proliferation of fibroblast cells (*p* < 0.05). It has been stated that incorporation of polysaccharides like chitosan or glycosaminoglycan into the proteins like collagen and gelatin not only increases their mechanical properties, but also improves their cellular activity (Chen et al. 2010; Mahboudi et al. 2015; Pezeshki-Modaress et al. 2013; Zhong et al. 2005). In this work from the in vitro study of HDF cells behavior on gelatin/chitosan nanofibers, it was established that the protein–polysaccharide blends and also nanofibrous morphology with high porosity favor cell attachment and proliferation by providing a three-dimensional extracellular environment. Advancement in fibroblast cells attachment to the fibers and also high proliferation rate on electrospun gelatin/chitosan scaffolds indicate high bioactive properties achieved with blending gelatin and chitosan in nanofibrous form and molecular interactions created between the gelatin, chitosan and HDF cell membrane.

## Conclusion

Based on our results, gelatin/chitosan nanofibrous scaffolds were fabricated using electrospinning at optimized condition with average fiber diameter in the range 180–196 nm. The fibrous morphologies of electrospun gelatin/chitosan scaffolds in culture medium were remained intact during 7 days. The electrospun gelatin/chitosan scaffolds have porosity around 92% and tensile strength of 1.1 MPa. The FTIR spectroscopy analysis demonstrated the presence of chitosan in nanofibrous structure. To assay the bioactivity of scaffolds, the attachment, morphology and proliferation of HDF cells on electrospun gelatin/chitosan scaffolds were analyzed. The morphological observation showed that HDF cells attached and spread well on gelatin/chitosan nanofibrous scaffolds exhibiting spindle-like shape. The SEM micrograph also revealed that cross-linked electrospun gelatin/chitosan blends were able to maintain their nanofibrous morphology in culture medium and provide steady physical and chemical support for cell growth. The MTS results demonstrated the significant beneficially influence of chitosan and also the nanofibrous morphological on the HDF cell proliferation. Considering the MTS assay, porosity and also the mechanical property of gelatin/chitosan nanofibers with different ratios, it could be stated that Chi 30 (70/30 gelatin/chitosan composition) has a good potential for using in skin tissue engineering application.
